# Aneurism Spontaneously Cured

**Published:** 1846-12

**Authors:** W. H. Robert

**Affiliations:** Madison, Ga.


*Aneurism, spontaneously cured.* By W. H. Robert, M.D., of Madison, Ga.—Mr T. Harris, aged about 35 years, was bled by a neighbor on the 17th April last. On seeing him a few days after, he stated that immediately after his arm was bound up he observed a tumor where he had been bled, which had continued to increase ever since. The arm now presented all the symptoms of aneurism; diminished temperature, indistinct pulsation of the radial artery at the wrist, a pulsating tumor which subsided on pressure, and filled again when this was discontinued, which ceased to pulsate when the brachial artery was compressed, and in which the bellows sound was distinctly heard.

I explained to Mr. H. the nature of the operation usual in such cases, and advised him to submit to it. Finding, however, that he was unwilling to adopt this course until he could gather in his crop, I advised him to keep the arm perfectly quiet. In July the tumor had attained the size of a hen's egg, but about the second week in August it began to diminish, the pulsation became less distinct in the tumor and stronger at the wrist, the arm grew warmer, and this state of things has been gradually progressing until the present time. The arm now presents the following appearance: it is as warm as the other; the pulsation at the wrist is normal; there is no pulsation whatever in the tumor, which is about the size of a filbert and apparently perfectly encysted, being easily moved about under the skin, and which does not diminish on pressure. I attribute this unexpected result to the quiescent state of the limb.—*Southern Med. & Surg. Jour.*

